# The Importance of the Coach in Predicting Implicit Beliefs about Skill and Beliefs about the Causes of Success in Handball Players

**DOI:** 10.3390/ijerph16010078

**Published:** 2018-12-29

**Authors:** Manuel Gómez-López, David Manzano-Sánchez, Juan Andrés Merino-Barrero, Alfonso Valero-Valenzuela

**Affiliations:** 1 Department of Physical Activity and Sport, Faculty of Sport Sciences, University of Murcia, Santiago de la Ribera, 30720 Murcia, Spain; david.manzano@um.es (D.M.-S.); avalero@um.es (A.V.-V.); 2 Health, Physical Activity and Education Research Group (SAFE-E0B5-04), University of Murcia, Santiago de la Ribera, 30720 Murcia, Spain; merinobarrero@gmail.com

**Keywords:** ability, coach, adolescents, incremental belief, entity belief

## Abstract

The objective of the present study was to determine the predictive capacity of the motivational climate generated by coaches and perceived by handball players on implicit beliefs about ability and beliefs about the causes of success in sport. The sample consisted of 444 youth handball players. These players completed the Beliefs about the Causes of Success in Sport Questionnaire, the Conceptions of the Nature of Athletic Ability Questionnaire, Version Two, and the Perceived Motivational Climate in Sport Questionnaire. The structural regression model showed that the mastery climate positively predicted the belief in incremental ability and that this in turn positively predicts both belief in athletic success through effort and ability. The results reflected the importance of the coach in the formative process of the player and the search for performance in sport.

## 1. Introduction

During the process of player training, organized and competitive sport becomes a context of abilities and achievements in which motivational factors have great relevance in terms of the long-term effects of sports practice on the practitioner’s psychosocial development. This process of sports training coincides with childhood and adolescence, stages at which young people are receptive to the influence of figures external to their sports practice. Roberts and Treasure argue that the athlete’s physical and psychological well-being depends on the social settings in which sports practice takes place, which is closely related to the coach’s role [1]. 

Specifically, in a team sport such as handball and with youth players, the coach is one of the main social agents [2] who has an essential role in the optimal development of the game [3,4], becoming one of the most important people in charge of promoting a positive training climate and influencing the way(s) players face the tasks proposed by the coach.

In order to understand the processes by which young people practise or abandon sports, we can use the social cognitive theory of goal perspectives [5]. The purpose of this theory is the analysis of the various dispositional and environmental factors that influence an athlete’s achievement motivation. According to this theory, athletes are driven by the need to show their competence and avoid demonstrating otherwise.

According to this theoretical construct, the dispositional factors (dispositional orientation) reflect the criterion by which athletes judge their level of competence and by which, subjectively, they define success or failure in sport. Studies have shown that goal orientations are good predictors of beliefs about the causes of success in sport [6,7,8]. Athletes with a mastery orientation judge their level of competence through comparison with themselves, perceive practice as an activity that reinforces the capacity for cooperation and increases the interest to learn. These athletes, therefore, believe that sports success is obtained thanks to efforts made and consider failure not as a negative and frustrating result but as an element for learning that always helps them to improve. In this way, they get a sense of pleasure, satisfaction and a greater commitment to the sports practice they carry out. Then there are athletes with an ego orientation. They judge their competence by demonstrating it, comparing themselves to and overcoming others; they perceive sports as a means to gain recognition and social status, and thus sporting success is achieved through ability and deception. This type of practice leads to boredom and a much lower level of intrinsic satisfaction, which implies a greater tendency to abandon sports practice, especially when these athletes question their own ability based on the small difficulties that arise in practising sport [9]. 

Another element affecting sports performance are situational factors, which refers to the set of signals generated by family, friends, coaches, etc., and perceived by the athlete in their environment, through which the keys to success and failure defined by Ames [10] as a motivational climate are identified. It should not be forgotten that sport is a medium of socializing influences that have repercussions on player training [11]. Depending on how the athlete perceives the context, there can be an ego-involving or performance motivational climate or a task-involving or mastery motivational climate [5,12]. The perception of a mastery motivational climate has been shown to be good for sports performance, enjoyment and satisfaction [3,13], and commitment to sport [4], and may enhance athletes’ psychological well-being by improving confidence and self-esteem, while also decreasing anxiety [14].

Bearing in mind the theory of self-determination [15], another important factor related to the motivation of adolescents in the field of sport are implicit beliefs about the ability to practice sports [16] which exert a strong influence on behaviour. Athletes can consider skill to be something that can be improved through learning, effort and training (incremental belief), or as innate and stable, and therefore independent of practice and effort (entity belief) [17,18]. Spray et al. [18] state that one can possess both incremental and entity implicit ability beliefs. This would mean that ability is influenced by an innate natural talent which can be modified through effort and training. Different studies have shown that incremental belief in ability is positively related to more self-determined forms of motivation, fun, persistence, and mastery- oriented goals, as if an athlete believes that his or her ability can improve, it is probable that he or she will enjoy sports practice more in the belief that positive results will be achieved. However, athletes with a belief that ability is stable will suffer from feelings of frustration and demotivation when satisfactory results are not achieved [17,19].

The importance of this study lies in the fact that no published papers have been found that have attempted to analyse the sum of the variables addressed here. Further relevancy lies in the importance of results for coaches when it comes to individualizing the treatment of their players. Therefore, the objectives of this study were two: (a) to describe the beliefs of the causes of success, implicit beliefs about ability and the perception of the motivational climate generated by the handball youth players’ coach and (b) to analyse the predictive capacity of the motivational climate perception generated by the coach in the implicit ability beliefs and beliefs about the causes of success in sports in youth handball players.

## 2. Methods

### 2.1. Participants 

Given the characteristics of the work, a non-experimental sampling system was not random but convenience-based. Four hundred and seventy-nine handball players from the juvenile category (250 boys and 229 girls) were selected to compete in the Spanish Autonomous Selections Championship (CESA). The age range was from 16 (40.1%) to 17 years (59.9%) (*M* = 216.60; *SD* = 0.50). These players were classified by the Higher Sports Council (CSD) as “High Performance Athletes” according to the Royal Decree 971/2007 [20], July 13, about high sportive people and high performance. These players did not appear in any list, nor were they published in the Official State Bulletin (BOE). The final total sample after excluding 33 players that were considered atypical for the statistical analysis or did not answer the entire questionnaire was 444 players (233 boys and 211 girls) with ages between 16 (41%) and 17 years (59%) (*M* = 16.59; *SD* = 0.49). According to the variable ‘years of experience as a federated handball player’, 85.6% of participants affirmed that they had more than 5 years of experience.

### 2.2. Measurement Instruments

This study employed the Beliefs about the Causes of Success in Sport Questionnaire (BACSSQ) [21,22]. We used the Spanish language version of this instrument [9], made up of 18 items which measure the perceptions of participants on effort (9 items), ability (4 items) and the use of deception techniques (5 items) for allowing them to achieve success in sport. The participants were asked: “What do you think people should do in order to be successful in the sport they practice most often?” The responses to this question were recorded on a Likert scale from (1) ‘totally disagree’ to (5) ‘totally agree’. In this study, the internal consistency of the subscales was α = 0.77, α = 0.73 and α = 0.84, respectively. The whole scale had a value of α = 0.79. 

Conceptions of the Nature of Athletic Ability Questionnaire-2 (CNAAQ-2) [17] was also employed in this study. We used the Spanish version of the scale [23], which is composed of 12 items divided into two higher order sub-scales called ‘incremental belief’ and ‘entity belief’. The entity belief sub-scale includes six items of which three correspond to the first order stable variable and the others to the talent variable. The incremental sub-scale includes six items of which three correspond to the first order improvement variable and the others to the learning variable. Participants are asked: “Your beliefs about your ability in sports are ...” The responses were recorded on a Likert scale from (1) ‘totally disagree’ to (5) ‘totally agree’. In the present study, Cronbach’s alpha values of 0.76, 0.86, 0.68 and 0.73 were respectively obtained. The total scale obtained a value of α = 0.79.

Lastly, Perceived Motivational Climate in Sport Questionnaire (PMCSQ-2) [12,24] was employed in this study. We used the Spanish version of this instrument [25], which is composed of 29 items divided into two dimensions that measure the ego-involving climate (14 items), called ‘Performance’ and the task-involving climate (15 items), called ‘Mastery’. Athletes were asked: “In my training group or team ...” and their responses were recorded on a Likert scale from (1) ‘totally disagree’ to (5) ‘totally agree’. In the present study, the internal consistency of the subscale mastery was α = 0.86 and performance was α = 0.85. The full scale obtained a value of α = 0.82.

### 2.3. Procedure

Permission was sought from the Royal Spanish Handball Association and the Handball Federation of Andalusia, which hosted the event, as well as from the various youth teams, in a letter which included an example of the research instrument and which set out the objectives of the study and how it was to be carried out. The data collection instruments were self-administered during the players’ free time in the different hotels where the teams were staying. Consent was obtained from the players and their parents or tutors and coaches. Participants were informed of the objective of the study, the voluntary nature of participation, that the data collected would be treated in the strictest confidence and that there were no right or wrong answers. Participants were asked to answer the questions as honestly as possible. The instrument took an average of about 30 minutes to administer. The ethical requirements relating to data collection were scrupulously respected and approval was obtained from the Ethics Committee of the Universidad de Murcia (ID: 1494/2017). Finally, it is worth highlighting that as well as the responses to the measurement instruments, gender, year of birth, years of experience as a handball player, playing position, and the numbers of hours per week dedicated to training, as well as the times it was carried out, were also recorded.

### 2.4. Data Analysis 

The descriptive statistics and the bivariate correlations of the variables considered in the study were calculated. Subsequently, a structural regression analysis was performed using a stepwise approach [26], with the objective of testing the relationships hypothesized between them. Finally, a multivariate analysis (MANOVA) was performed taking into account the variables of gender, playing position, number of hours dedicated to training each week, times at which training occurred, years of experience and their effect on the motivational climate promoted by the coach and beliefs regarding both skill and the causes of the players sports success. The effect size was calculated at the univariate level. Cohen [27] characterized the size of the effect as small (η2 = 0.01), medium (η2 = 0.06) and large (η2 = 0.13). All analyses were carried out with the SPSS 19.0 statistical package (IBM, Chicago, IL, USA) and Amos 19.0 (IBM, Chicago, IL, USA).

## 3. Results

### 3.1. Descriptive Analysis and Bivariate Correlations

The descriptive statistics (mean, standard deviation, asymmetry and kurtosis), Cronbach’s alpha values ​​for the subscales, as well as bivariate correlations for all variables studied are presented (Table 1). The data indicate a higher score in the perception of mastery climate, incremental belief and that success in sport is achieved through effort (*M* = 4.01, 4.35, 4.54, respectively), than in the case of performance climate, entity belief and belief that sports success is achieved through deception (*M* = 2.69, 2.46, 1.99, respectively). In the analysis of bivariate correlations, the variables under study were significantly related to each other (*p* < 0.01), excluding the performance climate with belief in effort and incremental belief with capacity belief.

### 3.2. Structural Regression Analysis

First, validation of the measurement model was carried out based on an analysis in which the latent variables freely correlated, dividing the items of each variable by pairs, so that half of the first items of each sub-scale were averaged so as to form the first block of items, and the second half of items were averaged to form the second block of items. Marsh [28] proposed the use of item pairs because their results are more reliable and tend to be distributed more normally; by reducing the number of observed variables, the model being identified with each latent variable was measured by at least two indicators [29].

Multivariate normality was verified by the Mardia coefficient (20.26) and considered to be adequate, as values lower than 70 in this index indicate that the departure from multivariate normality is not inappropriate for the analysis [30]. The assumption of multicollinearity was fulfilled since the bivariate correlations between the variables were below 0.85 [31]. The errors of the endogenous variables were independent because they were not correlated with other variables. The maximum likelihood method was used as the estimation method.

Taking into account that it is regarded as unwise to use a single global fit measure of the model, different absolute and relative adjustment indices were calculated [32]. As absolute indices, we used χ2 as well as the ratio between the χ2 / df index (degrees of freedom of the model). As to relative indices, the IFI (Incremental Fix Index), CFI (Comparative Fix Index) and TLI (Tucker-Lewis Index) were calculated. The RMSEA (Root Mean Square Error of Aproximation) and RMSR (Standarized Root Mean Square Residual) were also analysed [33]. The goodness of fit indices obtained were considered adequate, χ^2^ (19, *n* = 444) = 23.36; *p* < 0.00; χ^2^/ df = 1.63; CFI = 0.98; IFI = 0.98, TLI = 0.97; RMSEA = 0.03; SMSR = 0.03.

It was hypothesized that the perception of the climate generated by the coach would be a higher order factor that would act as a trigger for the implicit beliefs regarding skill, which in turn would predict the beliefs of the causes of the sporting success of handball players.

The Mardia coefficient (20.26) and the covariance matrix were used as input for the analysis of the data. The goodness of fit indices showed adequate values for the data [34], adjusting to the established parameters: χ^2^ (21, *n* = 444) = 21.14; *p* < 0.00, χ^2^/df = 2.07; CFI = 0.96; IFI = 0.93; RMSEA = 0.04; SMSR = 0.05. All relationships were significant and were examined through standardized regression weights.

**Figure 1:**
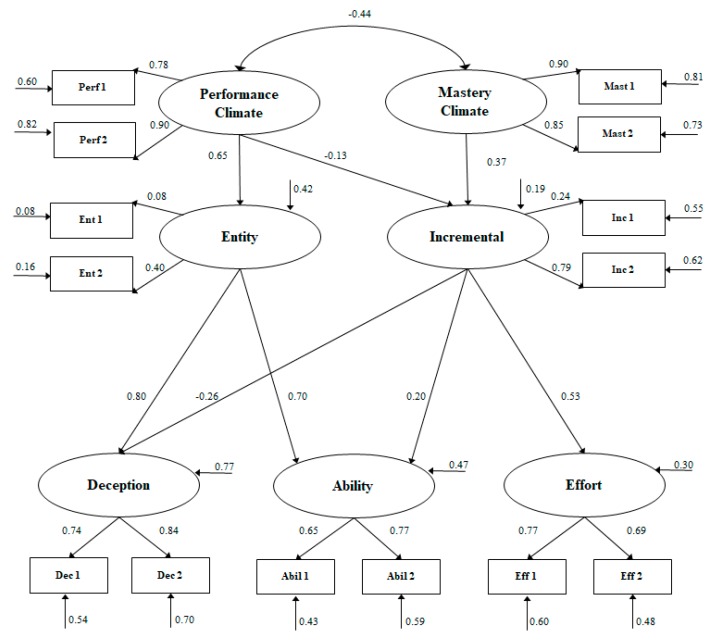
Structural regression model showing the relationships between the motivational climate promoted by the coach, belief in athletic ability and the beliefs about the causes of success in sports. All parameters are standardized and are statistically significant at *p* < 0.05. The explained variances are shown on the small arrows.

The results of this model (see Figure 1) show that both motivational climates correlated negatively with each other. Climate performance positively predicted entity belief (*β* = 0.65) and negatively predicted incremental belief (*β* = −13). By contrast, mastery climate positively predicted the belief in incremental ability (*β* = 0.37) without reflecting any prediction about entity belief. The entity belief positively predicted belief in the causes of sports success based on deception (*β* = 0.80) and ability (*β* = 0.70), without making any prediction about the belief in success in sport through effort. Finally, the belief in incremental ability positively predicted the belief in athletic success through effort (*β* = 0.53) and ability (*β* = 0.20) and negatively success based on deception (*β* = −0.26).

### 3.3. Multivariate Analysis

In order to analyse the relationships between the socio-demographic variables (gender, position, years of experience, number of weekly training sessions and number of hours of training per week) and dependent variables (variables included in the model of structural equations tested), a multivariate analysis of variance (MANOVA) was carried out. Significant differences at the multivariate level (see Table 2) were found only for gender (Pillai’s Trace = 0.039 (F_(1.339)_ = 2.26, *p* < 0.05)).

Subsequent univariate analyses showed significant differences between men and women for performance climate, with superior means in men, for beliefs in the causes of success in sports based on effort, with significantly higher scores in women, and deception techniques, in which men scored higher. The other variables analysed did not show statistical significance.

## 4. Discussion

In order to answer the objectives of the study, different statistical analyses were carried out (descriptive and correlation, multivariate and regression). The multivariate analysis showed that there were only statistically significant results in the gender variable, to the effect that the perception of performance climate was greater in men than in women. These results coincide in part with those found in other studies of team and individual sports practitioners, in which the perception of the performance climate was slightly higher in boys [11]. The results that refer to beliefs regarding the causes of success in sport showed significant differences in both effort and deception belief. It is demonstrated, therefore, that while women outnumber men in belief in effort, men score higher in belief in deception. These results coincide with those in Abraldes et al. [6] with swimmers and contradict those found by Ruiz-Juan et al. [35], in which there was a positive relationship between female canoeists and the belief that success in sport was achieved through skill and deception and boredom.

Unlike other studies, the multivariate analysis showed that there were no statistically significant results between beliefs regarding ability and the variable sex, coinciding with Wang and Biddle [19], and differing from the results provided by Li, Lee and Solmon [36], where significant differences between boys and girls were detected.

In order to explain the gender differences, there have been attempts to attend to social factors that influence the forms of sport socialization. It is believed that women understand sport as a cooperative activity oriented to leisure and recreation, whereas men tend to highlight the competitive element associated with sport [37,38,39]. In this case, because it is a very particular sample, based on a select group of players (players selections), it is very possible this could result in there not being any differences between boys and girls, and that they all have the same implicit beliefs about the ability level.

Regarding the main objective of this study, the results of the structural regression model show that mastery climate positively predicts belief in incremental ability and that this in turn positively predicts both the belief in sports success through effort and through ability. By contrast, performance climate positively predicts the belief of skill as an entity and this in turn predicts beliefs in ability and deception as causes of success in handball.

Regarding the limitations of this study, we recognize that in order to analyse the influence of the motivational climate on players of these ages, and following the literature review, it would be necessary to carry out long-term work with an interventional follow-up, in which not only the coach’s influence but also that of significant others, such as parents and peers, would be analysed. In this way, the interaction between the different agents in the creation of the motivational climate would be examined. Based on the results produced, training programs for parents and coaches could be designed to promote a better understanding of the importance they have in the training process of the players in order to develop an adequate motivational climate. Furthermore, it has been shown that the importance of significant others varies according to different parameters such as age, sex, the sport concerned and the competitive level of the sample. Therefore, the significance of results should be limited to the population represented in our sample.

A second limitation comes from the measurement of the perception of motivational climate, as observations or contrasts with records of attitudes of those involved have not been used, nor has it been contrasted with information that could have been provided by the coach him/herself [40]. 

We should admit as a third limitation the size of the analysed sample. In this study, 100% of the participants were the best players in the juveniles category at the national level and the number of participants was low and this limits the generalization of the results, as such, this study should be considered as preliminary. In the future, more replications are needed in other sport levels and other age ranges, covering all ages of adolescence.

Finally, as a fourth limitation, although the structural equation model presented is the one that presented the best fit, it is assumed that this model is only one of those possible [28]. Future studies could consider the variable ranking or position obtained at the end of the competition.

Therefore, in accordance with the results obtained, the importance of the figure of the coach in the formative process of the player and the pursuit of sports performance is once again highlighted. The study shows that a coach can pursue performance goals and victory with his or her players without having to orientate training sessions towards a performance climate. 

From the practical point of view, it is very important that the coach encourages a motivational mastery climate that enhances fun and satisfaction in the practice of sport, autonomy and competence, along with its psychological well-being and task orientation [41].

Thus, during training and competition the coach should consider each player’s mistakes as part of his or her learning and development and encourage effort, personal progress, skill development, cooperative learning and choice of tasks, always making sure that the player feels involved in the learning process [12,14].

## Figures and Tables

**Table 1:** Descriptive statistics and bivariate correlations of the sample.

Descriptive/Correlations		Range	*M*	*SD*	*A*	*K*	*α*	1	2	3	4	5	6	7
1.	Mastery	1–5	4.01	0.58	0.40	0.28	0.87	-	−0.38 **	−0.19 **	0.33**	0.24**	−0.03	−0.29 **
2.	Performance	1–5	2.69	0.71	0.06	−0.21	0.91	-	-	0.24 **	−0.24**	−0.08	0.26 **	0.47 **
3.	Entity	1–5	2.46	0.75	−0.99	−0.31	0.87	-	-	-	−0.31**	−0.19 **	0.27 **	0.31 **
4.	Incremental	1–5	4.35	0.58	−0.98	0.16	0.83	-	-	-	-	0.39 **	−0.01	−0.31 **
5.	Effort	1–5	4.54	0.50	−1.14	0.81	0.71	-	-	-	-	-	0.16 **	−0.21 **
6.	Ability	1–5	3.42	0.91	−0.10	−0.55	0.72	-	-	-	-	-	-	0.35 **
7.	Deception	1–5	1.99	0.84	0.72	−0.16	0.73	-	-	-	-	-	-	-

Note: ** *p* < 0.01; *M* = Mean; *SD* = Standard Deviation; *A* = Asymmetry; *K* = Kurtosis; *α* = Cronbach alpha.

**Table 2:** Univariate analysis of the variance for gender as a function of the perceived motivational climate, the beliefs of the causes of success in sport and the perception of ability.

Univariate Analysis	Males		Females		ANOVAS		
	*M*	*SD*	*M*	*SD*	*F*	*p*	*η*2
1. Mastery	3.93	0.62	4.10	0.53	3.44	0.06	0.00
2. Performance	2.80	0.69	2.57	0.73	8.05	0.00 **	0.02
3. Effort	4.47	0.55	4.63	0.44	6.08	0.01 **	0.02
4. Ability	3.56	0.89	3.28	0.92	1.72	0.19	0.01
5. Deception	2.17	0.88	1.81	0.76	8.04	0.00 **	0.02
6. Entity	2.55	0.78	2.36	0.72	0.68	0.40	0.00
7. Incremental	4.28	0.62	4.43	0.55	1.28	0.26	0.00

Note: ** *p* < 0.01; *M* = Mean; *SD* = Standard Deviation.
